# Impact of birth defect prevention and control programs on mortality among children with birth defects from 2012 to 2023 in Shenzhen, China

**DOI:** 10.3389/frhs.2025.1657703

**Published:** 2025-09-17

**Authors:** Xueyu Yang, Guanglin Zhao, Jing Zheng, Shuyan Jin

**Affiliations:** ^1^ Women's and Children’s Information Department, Shenzhen Maternity and Child Healthcare Hospital, Shenzhen, Guangdong, China; ^2^ Shenzhen Health Development Research and Data Management Center, Shenzhen, Guangdong, China; ^3^ Health Department, Shenzhen Maternity and Child Healthcare Hospital, Shenzhen, Guangdong, China

**Keywords:** birth defects, prevention and control programs, mortality, evaluation, Shenzhen

## Abstract

**Background:**

Birth defects are an important cause of fetal and neonatal mortality and represent a major global public health concern. Shenzhen has implemented several prevention and control programs in recent years. However, the effectiveness in reducing mortality among affected children has not been systematically evaluated.

**Objective:**

To assess the impact of birth defect prevention and control programs on mortality among children with birth defects in Shenzhen from 2012 to 2023, and to provide evidence for program evaluation and maternal–child health policy development.

**Methods:**

All registered cases of children with birth defects in Shenzhen between 2012 and 2023 were included. The study period was divided into three phases according to program implementation: Phase I (2012–2017), Phase II (2018–2021), and Phase III (2022–2023). Mortality outcomes included early fetal death, late fetal death, and early neonatal death. Trends were analyzed using the Cochran-Armitage test with Bonferroni-adjusted pairwise comparisons. Multivariable logistic regression adjusted for confounders and subgroup analyses were conducted by maternal household registration status (local vs. non-local).

**Results:**

From Phase I to Phase III, early fetal mortality increased (26.1% vs. 29.7% vs. 33.4%), whereas late fetal mortality (5.7% vs. 4.1% vs. 3.6%) and early neonatal mortality (1.0% vs. 0.5% vs. 0.3%) declined significantly(*P* < 0.001 for trends). Logistic regression showed lower risks of late fetal and early neonatal death in Phases II and III compared with Phase I, with greater reductions among children of non-local mothers.

**Conclusion:**

Birth defect prevention and control programs in Shenzhen were associated with reduced late fetal and early neonatal mortality, especially in non-local populations, Providing evidence to guide maternal-child health policy.

## 1 Introduction

Birth defects, also known as congenital anomalies, are structural or functional abnormalities (1) from genetic or environmental factors during fetal development. Over 8,000 types of birth defects have been identified and are classified by anatomical system (2), structural/functional characteristics (3) or morphological (4). They are a leading cause of early miscarriage, stillbirth, infant mortality and childhood mortality, and congenital disability (5). In China, approximately 5.6% of newborns are affected annually (6), and national surveillance in 2019 reported 5.7% mortality rate among affected children, including 4.1% stillbirths and 1.7% postnatal deaths (7). Birth defects significantly compromise survival and quality of life while imposing heavy society and families burdens, making them a major public health concern (8–11).

To address this, the Chinese government has strengthened prevention through major public health initiatives, including free preconception health exams, folic acid supplementation, thalassemia control, and neonatal screening in impoverished areas (12). Key policy documents, such as the *National Comprehensive Prevention and Control Plan for Birth Defects* (13) and the *Capacity-Building Plan for Birth Defect Prevention and Control (2023–2027)* (14), have established of a three-tiered prevention system: (1) primary prevention via premarital and pre-pregnancy healthcare; (2) secondary prevention through standardized prenatal screening and diagnosis; and (3) tertiary prevention via neonatal screening to reduce congenital disabilities.

**Figure 1:**
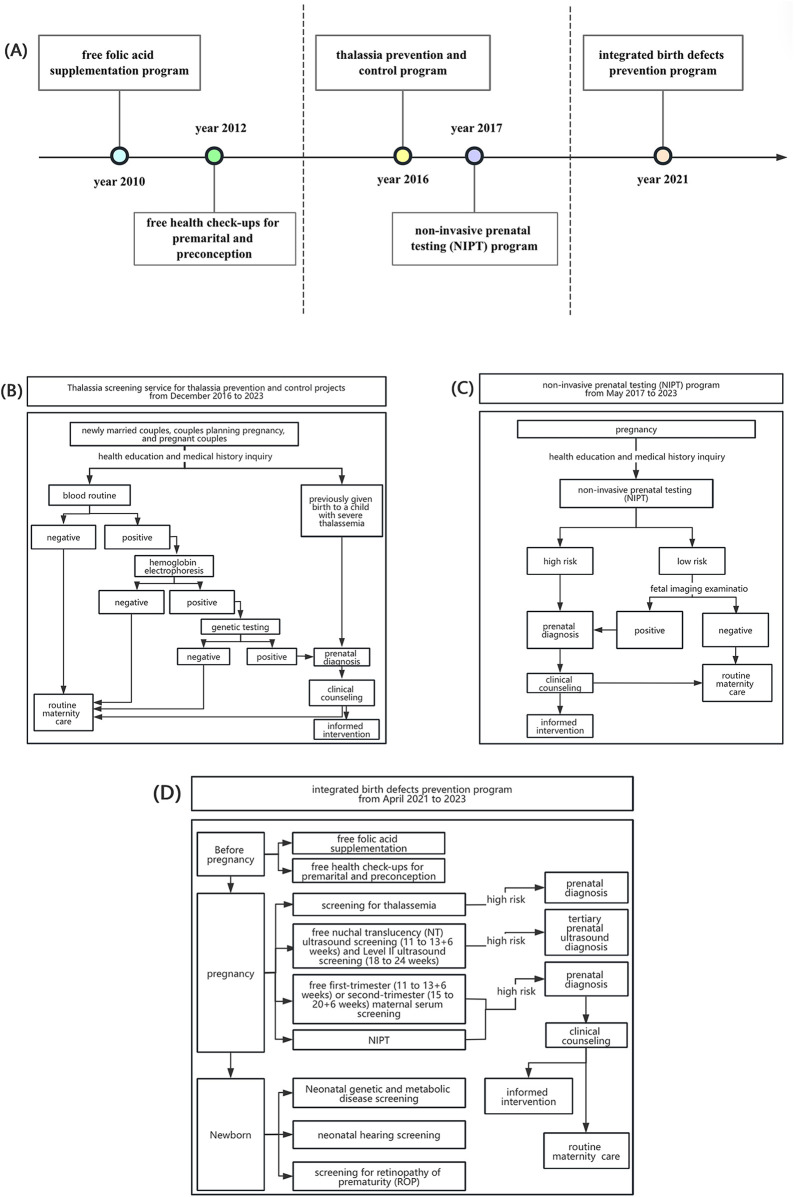
Introduction of birth defect prevention and control programs in Shenzhen from 2012 to 2023. **(A)** Timeline of birth defect prevention and control programs in Shenzhen during 2010–2023. **(B)** The detailed service workflow of thalassemia prevention and control program. **(C)** The detailed service workflow of non-invasive prenatal testing (NIPT) program. **(D)** The detailed service workflow of integrated birth defects prevention program.

As a Special Economic Zone, Shenzhen has integrated national strategies into its public health agenda and, over the past decade, has implemented a series of population-based interventions (Figure 1A).

Since 2010, Shenzhen has introduced a series of programs to prevent birth defects. A free folic acid supplementation program was launched to reduce neural tube defects, followed in 2012 by complimentary premarital and preconception health examinations (15), forming the foundation for early-stage prevention.

In 2016, the city initiated a comprehensive thalassemia prevention program offering free education, screening, genetic testing, and counseling to newly eligible couples (16). The detailed service workflow is illustrated in Figure 1B. In 2017, Shenzhen became the first city in China to implement a population-based non-invasive prenatal testing (NIPT) program, providing free high-throughput sequencing for trisomies 21, 18, and 13 (17), with follow-up counseling and prenatal decision support (Figure 1C).

By 2021, these measures were integrated into a comprehensive program covering women with Guangdong household registration, spouses of registrants, and those with valid residence permits (Figure 1D). Services expanded to include folic acid supplementation, preconception examinations, thalassemia prevention, free nuchal translucency (NT) and Level II ultrasound screening, maternal serum testing for Down syndrome, subsidies for tertiary diagnostic testing in high-risk cases (18), and free screening for metabolic disorders, hearing loss, and retinopathy of prematurity.

These initiatives mark a shift from disease-specific to life-course prevention, embedding interventions across premarital, preconception, prenatal, and neonatal stages. As a result of these initiatives, the coverage rate of thalassemia screening among pregnancy couples in Shenzhen increased from 11% before 2017 to 88% in 2023, the NIPT coverage rate rose from 20% to 93%, and the prenatal fetal structural anomaly screening coverage rate reached 96%—data from Shenzhen Maternal and Child Health Information System (MCHIS). Despite these gains, their impact on health outcomes, particularly mortality among affected children, remains underexplored. Existing studies have emphasized process indicators, excluded fetal deaths before 28 weeks, and often overlooked disparities related to maternal household registration (19–22)—a key determinant in Shenzhen, where migrants comprise 65.9% of the population and face barriers to maternal health services.

Globally, early neonatal deaths account for over 70% of neonatal mortality, with congenital anomalies and prematurity remaining leading causes, especially in high-income settings where declines have been modest (23–25). Thus, assessing perinatal and early neonatal mortality provides meaningful insight into the potential impact of birth defect prevention programs, which specifically target these major contributors.

This study uses data spanning 2012–2023 from MCHIS to assess the impact of these programs on early fetal, late fetal, and early neonatal mortality, providing evidence to guide maternal and child health policy in China.

## 2 Methods

### 2.1 Study population

The study population comprised all children with birth defects who were delivered in Shenzhen hospital between 2012 and 2023, including cases diagnosed prenatally or within the first year after birth (encompassing stillbirths, fetal deaths, and live births). Cases involving minor abnormalities or clinical variants (e.g., pericardial effusion, pulmonary hypertension, twin-to-twin transfusion syndrome) and metabolic disorders in neonates were excluded from the registry.

Surveillance data included demographic information of the mother and child, pregnancy outcomes, and details of birth defect diagnosis and classification. Demographic variables were sourced from prenatal health records in the SMCHIS, while diagnostic and outcome data were extracted from clinical documentation. Diagnoses of birth defects were based on the International Classification of Diseases, 10th Revision (ICD-10) (26). Cases were registered within three days of diagnosis by healthcare professionals through the SMCHIS. The study protocol was approved by the Ethics Committee of Shenzhen Maternity and Child Healthcare Hospital (Approval No. SFYLS [2024]110). Informed consent was waived because the research utilized fully anonymized, retrospective administrative data, and involved no more than minimal risk to the subjects and using these data did not adversely affect the rights or welfare of the participants.

### 2.2 Data quality

Data quality was ensured through a multi-tiered audit process: obstetric institutions, district-level maternal and child health hospitals, and the municipal maternal and child health hospital conducted monthly, quarterly, and annual reviews to validate data accuracy and completeness, respectively, based on laboratory reports and medical records.

### 2.3 Division of research stages

The study period was 2012–2023. At the outset, two foundational programs—the free folic acid supplementation program and the free premarital and preconception health check-ups program—had already been implemented and continued throughout the entire study period. These ongoing measures were therefore not the focus of our phase-specific analysis. Given the close initiation timelines of the Thalassemia Prevention and Control Program (December 2016) and the Non-Invasive Prenatal Testing (NIPT) Program (May 2017), together with the typical lag time required for policy uptake and full implementation, we divided the study period into three distinct phases:

- Phase I (2012–2017): Foundational Stage, characterized by the initial implementation of core preventive measures (folic acid, premarital/preconception checks).
- Phase II (2018–2021): Expansion Phase, marked by the scaling up of comprehensive screening programs (thalassemia prevention, NIPT).
- Phase III (2022–2023): Integration and Enhancement Phase, defined by the launch of the 2021 Comprehensive Birth Defect Prevention Initiative, integrating new services (e.g., free level II ultrasound, monogenic disease screening/diagnosis).

**Figure 2:**
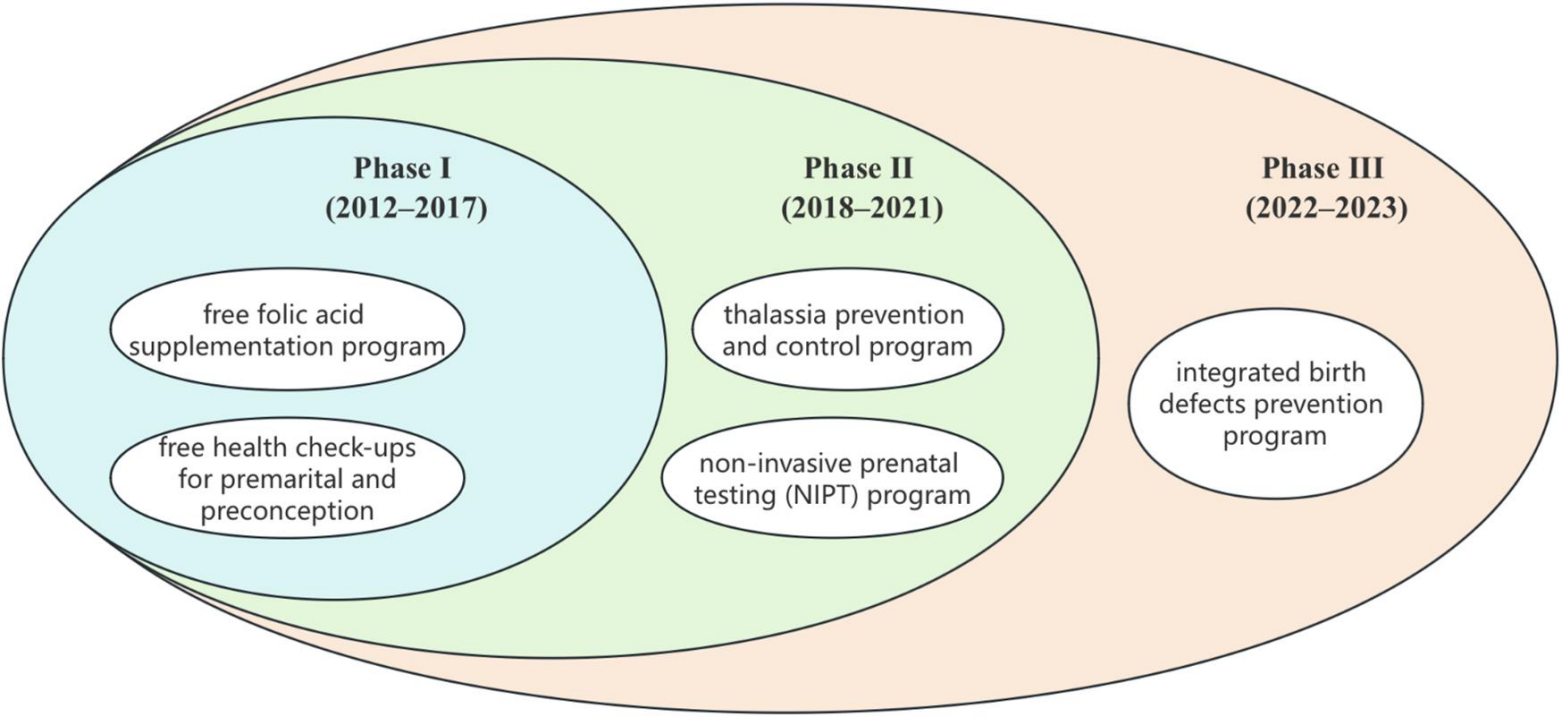
Sequential and overlapping implementation of birth defect prevention and control programs in Shenzhen.

Because these programs were introduced sequentially and overlapped in practice, the study design does not aim to isolate the independent effect of each single program. Instead, our analytic framework treats each phase as a cumulative package of programs, reflecting the real-world evolution of Shenzhen's programs. The overlapping nature of these interventions is illustrated in Figure 2.

### 2.4 Characteristic indicators

The study included several variables reflecting the characteristics of children with birth defects and their mothers: maternal age, maternal education level, maternal household registration type, parity, plurality, number of antenatal visits, maternal comorbidities, obstetric complications and pregnancy outcome. Given that maternal age ≥35 years is an indication for invasive prenatal testing (27), maternal age was categorized as <35 years and ≥35 years. Education level was classified as low (high school or below) and high (college or above). Maternal household registration type (hukou) was categorized as local-registered and non-local-registered. Plurality was defined as singleton (1 fetus) or multiple (>1 fetus). Parity was categorized as nulliparous (0 prior deliveries) or multiparous (≥1 prior delivery). Number of antenatal visits was categorized as <5 times vs. ≥5 times according to the national standard in China. Maternal comorbidities include hypertension, preeclampsia, diabetes, and major chronic diseases. Obstetric complications include such as placental abruption, premature rupture of membranes, and preterm delivery. Pregnancy outcomes were classified as live birth or fetal death/stillbirth. Birth defect cases were categorized into four subgroups based on congenital anomaly characteristics: (1) isolated structural anomalies (ISA); (2) multiple congenital anomalies (MCA); (3) chromosomal abnormalities/genetic abnormalities(C/GAs); and (4) other congenital anomalies (OCA). ISA included defects involving the central nervous system, cardiovascular system, gastrointestinal tract, genitourinary system, musculoskeletal system/other organs, and orofacial clefts. MCA was defined as the presence of two or more unrelated anomalies across different organ systems. C/GAs included chromosomal disorders such as polyploidy, aneuploidy, and gene disorders such as thalassemia. OCA encompassed congenital anomalies not included in the previous three groups (19).

### 2.5 Outcome measures

The primary outcome measures were early fetal death, late fetal death, and early neonatal death. In accordance with WHO-defined Perinatal Period I criteria, which is adopted by China, perinatal mortality includes stillbirths from 28 gestational weeks to neonatal deaths within 7 days (8). Therefore, in this article, fetal deaths were classified as early fetal death (fetal deaths <28 gestational weeks) and late fetal death (fetal deaths ≥28 gestational weeks). Early neonatal death was defined as death occurring between 0 and 6 days after birth.

### 2.6 Statistical analysis

Categorical variables were summarized as frequencies and percentages. Mortality rates with 95% confidence intervals (CIs) were estimated for each study phase. Trends across phases were evaluated using the Cochran–Armitage test, with subgroup analyses by maternal household registration. Pairwise comparisons were performed using the Wilcoxon rank-sum test (Mann–Whitney *U* test) with Bonferroni correction. Multivariable logistic regression assessed associations between study phases and mortality outcomes, adjusting for maternal household registration, maternal age, maternal education level, parity and plurality. Subgroup analyses by maternal household registration also applied Bonferroni correction. As missing data covariates missingness less than 10%, no imputation was conducted. All statistical analyses were performed in R software (v4.3.0), with two-sided *P* value < 0.05 was considered statistically significant.

## 3 Results

### 3.1 Basic characteristics of children with birth defects and their mothers

The basic characteristics of children with birth defects and their mothers across the three study phases are summarized in Table 1. From 2012 to 2023, a total of 87,858 cases were registered in Shenzhen, including 41,056 in Phase I, 31,818 in Phase II, and 14,984 in Phase III.

**Table 1:** Basic characteristics of children with birth defects and their mothers.

Variables	Phase I (2012–2017)	Phase II (2018–2021)	Phase III (2022–2023)	*P* for trend
	(*N* = 41,056)	(*N* = 31,818)	(*N* = 14,984)	
Maternal household registration				<0.001
Local-registered	31,323 (76.3%)	21,057 (66.2%)	9,127 (60.9%)	
Non-local-registered	9,733 (23.7%)	10,761 (33.8%)	5,857 (39.1%)	
Maternal education level				<0.001
High school or below	33,591 (81.8%)	21,569 (67.8%)	7,984 (53.3%)	
College or above	7,446 (18.1%)	10,248 (32.2%)	7,000 (46.7%)	
Maternal age				<0.001
<35 yrs	35,328 (86.0%)	26,229 (82.4%)	11,849 (79.1%)	
≥35 yrs	5,727 (13.9%)	5,589 (17.6%)	3,135 (20.9%)	
Plurality				0.037
Singleton	38,057 (92.7%)	30,189 (94.9%)	14,153 (94.5%)	
Multiple	2,030 (4.9%)	1,629 (5.1%)	831 (5.5%)	
Parity				<0.001
Nulliparous	21,220 (51.7%)	15,034 (47.3%)	6,954 (46.4%)	
Multiparous	18,927 (46.1%)	16,657 (52.4%)	8,030 (53.6%)	
Antenatal visits				<0.001
<5	29,109 (70.9%)	22,319 (70.1%)	10,980 (73.3%)	
≥5	11,947 (29.1%)	9,499 (29.9%)	4,004 (26.7%)	
Maternal comorbidities				<0.001
No	38,437 (93.6%)	28,573 (89.8%)	12,898 (86.1%)	
Yes	2,619 (6.4%)	3,245 (10.2%)	2,086 (13.9%)	
Obstetric complications				<0.001
No	35,707 (87.0%)	26,858 (84.4%)	12,904 (86.1%)	
Yes	5,349 (13.0%)	4,960 (15.6%)	2,080 (13.9%)	
Outcome of the birth				<0.001
Live birth	28,036 (68.3%)	21,067 (66.2%)	9,447 (63.0%)	
Fetal death/stillbirth	13,020 (31.7%)	10,751 (33.8%)	5,537 (37.0%)	
Birth defect type				
ISA	31,747 (77.3%)	22,732 (71.4%)	9,905 (66.1%)	<0.001
MCA	3,997 (9.7%)	3,442 (10.8%)	1,623 (10.8%)	<0.001
C/GAs	2,269 (5.5%)	3,561 (11.2%)	2,530 (16.9%)	<0.001
OCA	3,043 (7.4%)	2,083 (6.5%)	926 (6.2%)	0.031

Over time, the sociodemographic profile of mothers changed markedly. The proportion of non-local-registered mothers increased steadily from 23.7% in Phase I to 39.1% in Phase III (*P* for trend < 0.001). Similarly, the proportion of mothers aged ≥35 years rose from 13.9% to 20.9% (*P* for trend <0.001), while maternal education levels improved substantially, with college or above rising from 18.1% to 46.7% (*P* for trend <0.001). Reproductive characteristics also shifted. Multiparous mothers increased from 46.1% to 53.6% (*P* for trend <0.001), and multiple gestations rose slightly from 4.9% to 5.5% (*P* = 0.037), although the latter change was small in magnitude. The proportion of women with ≥5 antenatal visits decreased modestly over time (29.1%–26.7%, *P* for trend <0.001). Regarding maternal health status, comorbidities became more prevalent (6.4%–13.9%, *P* for trend <0.001), as did obstetric complications (13.0% to 13.9%, *P* for trend <0.001). Correspondingly, the proportion of fetal deaths or stillbirths increased from 31.7% to 37.0% (*P* for trend <0.001). The distribution of birth defect types also shifted. Isolated structural anomalies decreased from 77.3% to 66.1% (*P* for trend <0.001), while chromosomal/genetic anomalies rose markedly from 5.5% to 16.9% (*P* for trend <0.001). Multiple congenital anomalies also showed a modest but significant increase (9.7% to 10.8%, *P* for trend <0.001), whereas other congenital anomalies declined slightly (7.4% to 6.2%, *P* = 0.031).

Together, these findings highlight substantial temporal changes in maternal demographics, pregnancy characteristics, and the spectrum of birth defects, which should be considered when interpreting mortality trends across phases.

### 3.2 Mortality rates and trends among children with birth defects

**Figure 3:**
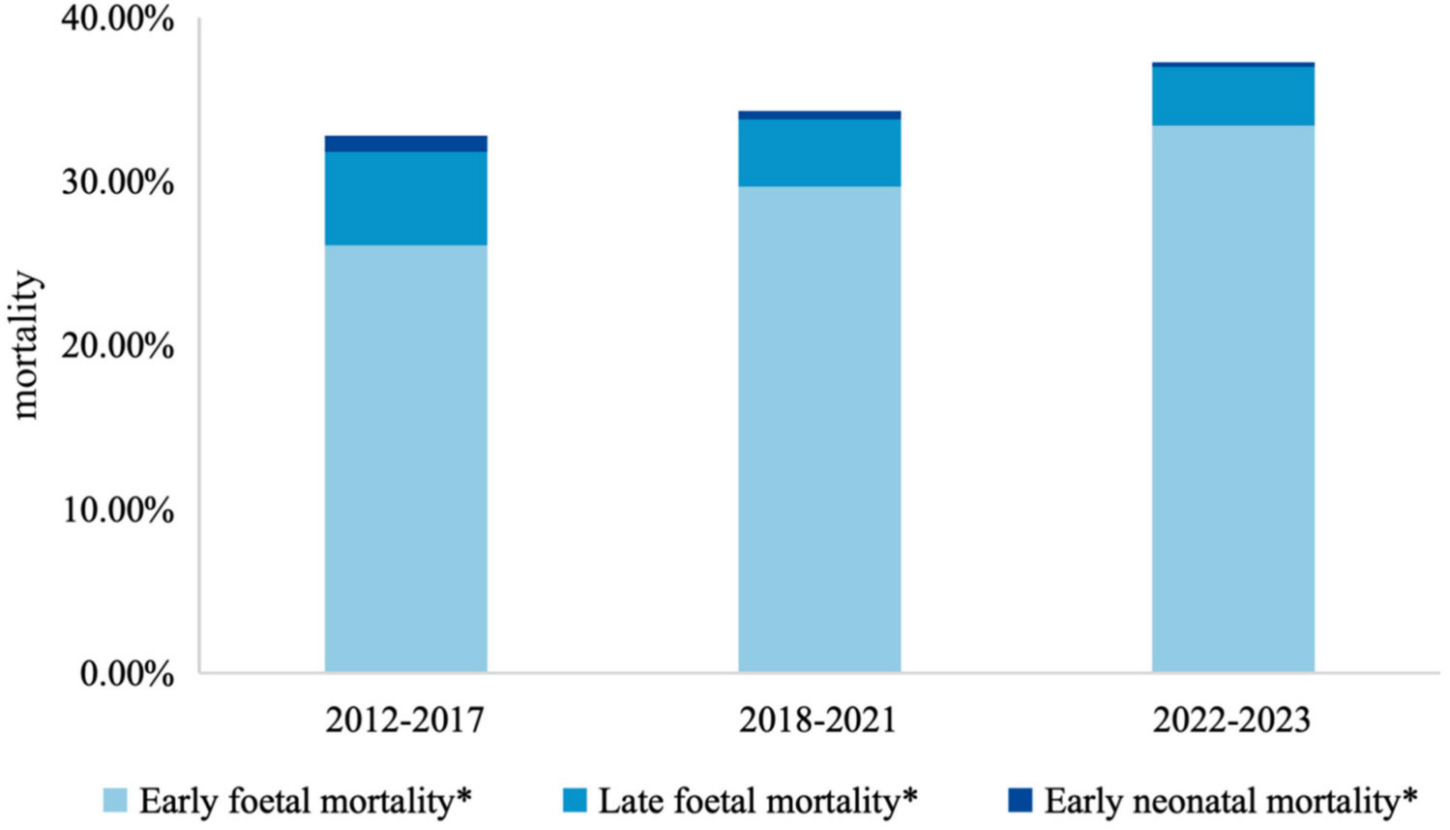
Trends on mortality among children with birth defects from 2012 to 2023. *: *P* value of Cochran-Armitage Test <0.001.

Figure 3 and Table 2 summarize mortality rates and their temporal trends across the three phases. Overall, early fetal death increased significantly from 26.1% (95% CI: 25.6–26.5) in Phase I to 33.4% (95% CI: 32.6–34.1) in Phase III (*P* for trend <0.001), with significant differences across both transitions. In contrast, late fetal death decreased from 5.6% (95% CI: 5.4–5.9) to 3.6% (95% CI: 3.3–3.9) (*P* for trend <0.001) and early neonatal death showed a consistent downward trend, from 1.0% (95% CI: 0.9–1.1) in Phase I to 0.3% (95% CI: 0.3–0.4) in Phase III (*P* for trend <0.001), with reductions observed between both Phase I and II and Phase II and III.

**Table 2:** Mortality rates and 95%CI among children with birth defects.

Outcomes	All	Birth defect type				Maternal household registration	
		ISA	MCA	C/GAs	OCA	Local-registered	Non-local-registered
Early fetal death							
Phase I	26.1% (25.6–26.5)	18.3% (17.8–18.7)	45.6% (44.1–47.2)	73.1% (71.3–74.9)	46.9% (45.1–48.6)	27.2% (26.3–28.1)	25.7% (25.2–26.2)
Phase II	29.7% (29.2–30.2)	19.3% (18.8–19.8)	44.8% (43.1–46.4)	74.3% (72.8–75.7)	42.2% (40.1–44.4)	28.2% (27.3–29.0)	30.5% (29.9–31.1)
Phase III	33.4% (32.6–34.1)	20.1% (19.3–20.9)	48.6% (46.1–51.0)	73.1% (71.4–74.8)	40.2% (37.1–43.4)	30.6% (29.4–31.8)	35.2% (34.2–36.1)
*P* ^a^	<0.001	<0.001	0.135	0.970	<0.001	<0.001	<0.001
*P* ^b^	<0.001	0.007	1	0.977	0.003	0.363	<0.001
*P* ^c^	<0.001	0.287	0.035	0.938	0.86	0.003	<0.001
Late fetal death							
Phase I	5.6% (5.4–5.9)	4.3% (4.1–4.5)	11.6% (10.6–12.6)	9.0% (7.9–10.3)	9.6% (8.6–10.7)	3.9% (3.5–4.3)	6.2% (5.9–6.5)
Phase II	4.1% (3.9–4.3)	2.8% (2.6–3.0)	7.4% (6.6–8.3)	8.5% (7.7–9.5)	5.4% (4.5–6.4)	3.1% (2.8–3.5)	4.6% (4.3–4.9)
Phase III	3.6% (3.3–3.9)	2.0% (1.7–2.3)	8.9% (7.6–10.4)	6.4% (5.5–7.4)	3.9% (2.8–5.3)	3.5% (3.0–4.0)	3.7% (3.3–4.1)
*P* ^a^	<0.001	<0.001	<0.001	0.001	<0.001	0.072	<0.001
*P* ^b^	<0.001	<0.001	<0.001	1	<0.001	0.008	<0.001
*P* ^c^	0.029	<0.001	0.196	0.005	0.244	0.66	<0.001
Early neonatal death							
Phase I	1.0% (0.9–1.1)	0.7% (0.7–0.8)	2.4% (1.9–2.9)	0.5% (0.3–0.9)	2.2% (1.7–2.8)	0.5% (0.4–0.7)	1.1% (1.0–1.3)
Phase II	0.5% (0.5–0.6)	0.4% (0.3–0.5)	1.3% (1.0–1.8)	0.2% (0.1–0.4)	1.0% (0.6–1.5)	0.3% (0.2–0.4)	0.7% (0.6–0.8)
Phase III	0.3% (0.3–0.4)	0.3% (0.2–0.5)	0.7% (0.4–1.2)	0.0% (0.0–0.2)	0.5% (0.2–1.3)	0.3% (0.2–0.4)	0.4% (0.3–0.5)
*P* ^a^	<0.001	<0.001	<0.001	<0.001	<0.001	0.007	<0.001
*P* ^b^	<0.001	<0.001	<0.001	0.057	<0.001	0.014	<0.001
*P* ^c^	0.034	0.780	0.258	0.278	0.566	1	0.029

^a^ *P* value for Cochran-Armitage Test.

^b^ *P* value for Bonferroni correction between Phase I and Phase II.

^c^ *P* value for Bonferroni correction between Phase II and Phase III.

When stratified by defect type, patterns differed. For isolated structural anomalies, early fetal death rose modestly from 18.3% to 20.1%, though the Phase II–III increase was not statistically significant (*P*^c^ = 0.287). Late fetal death declined steadily (4.3%–2.0%), and early neonatal death decreased to 0.3%, with no further decline after Phase II. For multiple congenital anomalies, early fetal death increased in Phase III (45.6% to 48.6%, *P*^c^ = 0.035), while both late fetal death and early neonatal death decreased, with the sharpest declines occurring in Phase II. For chromosomal/genetic anomalies, early fetal death remained persistently high (≈73%) with no meaningful temporal change, whereas late fetal death declined in Phase III (*P*^c^ = 0.005) and early neonatal death showed only a modest decrease.

Stratification by maternal household registration revealed further heterogeneity. In Phase I, early fetal death was slightly lower among non-local mothers compared with local mothers (25.7% vs. 27.2%), but the increase over time was greater among non-local populations (an increase of 9.5 percentage points vs. 3.4 percentage points in locals by Phase III). By contrast, reductions in late fetal and early neonatal deaths were more substantial among non-local groups (declines of 2.5 and 0.7 percentage points, respectively), suggesting differential temporal patterns across populations.

### 3.3 Multivariable logistic regression analysis

Table 3 presents results from multivariable logistic regression. After adjusting for maternal demographic and obstetric factors, early fetal death showed significantly increased odds in Phase II (AOR = 1.31, 95% CI: 1.27–1.35) and Phase III (AOR = 1.56, 95% CI: 1.50–1.63) compared with Phase I. Although the absolute increase in early fetal death was modest, an odds ratio above 1.5 in Phase III suggests a clinically meaningful elevation in risk that warrants attention in perinatal care practices. In contrast, late fetal death was less likely in later phases, with reductions in both Phase II (AOR = 0.74, 95% CI: 0.69–0.79) and Phase III (AOR = 0.67, 95% CI: 0.61–0.73). These effect sizes indicate an approximately 25%–33% lower risk, which is of public health importance given the burden of late fetal mortality. Early neonatal death also declined substantially, with nearly a 40% reduction in Phase II (AOR = 0.61, 95% CI: 0.51–0.72) and over 50% in Phase III (AOR = 0.47, 95% CI: 0.35–0.61).

**Table 3:** Multivariable logistic regression analysis between study phases and mortality outcomes.

Outcomes	All^a^		Local-registered^b^		Non-local-registered^b^		*P* for interaction^c^
	AOR (95% CI)	*P*	AOR (95% CI)	*P*_adjust_	AOR(95% CI)	*P*_adjust_	
Early fetal death							
Phase I	Reference		Reference		Reference		
Phase II	1.309 (1.265–1.354)	<0.001	1.069 (0.993–1.149)	0.225	1.673 (1.596–1.753)	<0.001	<0.001
Phase III	1.564 (1.499–1.631)	<0.001	1.229 (1.127–1.340)	<0.001	2.112 (1.985–2.249)	<0.001	<0.001
Late fetal death							
Phase I	Reference		Reference		Reference		
Phase II	0.741 (0.691–0.793)	<0.001	0.827 (0.713–0.959)	0.035	0.709 (0.655–0.768)	<0.001	0.246
Phase III	0.668 (0.606–0.734)	<0.001	1.025 (0.860–1.219)	1	0.604 (0.535–0.680)	<0.001	<0.001
Early neonatal death							
Phase I	Reference		Reference		Reference		
Phase II	0.608 (0.510–0.722)	<0.001	0.576 (0.369–0.884)	0.039	0.597 (0.492–0.720)	<0.001	0.617
Phase III	0.465 (0.350–0.606)	<0.001	0.747 (0.430–1.247)	0.839	0.403 (0.286–0.553)	<0.001	0.243

^a^ Model 1 adjusting for maternal household registration, maternal age, maternal education level, parity, plurality, antenatal visits, maternal comorbidities and obstetric complications.

^b^ Model 2 adjusting for maternal age, maternal education level, parity and plurality, antenatal visits, maternal comorbidities and obstetric complications.

^c^ Model 3 adds the interaction term of mother's household registration type and time stage on the basis of model 1.

***P***_adjust_: *P* value of Bonferroni correction for multiple testing across subgroup analyses.

The interaction analyses confirmed that these temporal patterns differed significantly between local and non-local populations for early and late fetal death (*P* for interaction <0.001), but not for early neonatal death, suggesting that changes in mortality patterns over time were unequally distributed across population subgroups. Stratified analyses revealed heterogeneity by maternal household registration. Among local-registered mothers, the risk of early fetal death rose modestly in Phase III (AOR = 1.23, 95% CI: 1.13–1.34), a small but notable increase that may still carry clinical relevance. Late fetal death showed a transient reduction in Phase II but not in Phase III, while early neonatal death declined in Phase II (AOR = 0.58, 95% CI: 0.37–0.88), corresponding to about a 40% reduction, though the Phase III association was attenuated and no longer significant. In contrast, among non-local-registered mothers, early fetal death increased more sharply across both transitions (Phase II: AOR = 1.67, 95% CI: 1.60–1.75; Phase III: AOR = 2.11, 95% CI: 1.99–2.25). Meanwhile, both late fetal death and early neonatal death consistently declined, with Phase III showing the strongest reductions (late fetal death: AOR = 0.60, 95% CI: 0.54–0.68; early neonatal death: AOR = 0.40, 95% CI: 0.29–0.55).

## 4 Discussion

Based on data from MCHIS, this study evaluated the impact of these programs on mortality among affected children. The results demonstrated a consistent decline in late fetal and early neonatal mortality rates as the prevention programs progressed, although trends varied across different types of birth defects. Multivariable logistic regression analyses, adjusting for potential confounders, yielded consistent findings. These changes may be attributed to several factors. The enhanced screening programs, such as advanced techniques like NIPT and expanded ultrasound, may improved the detection rate of severe fetal birth defects earlier in gestation (28, 29). This enabled more comprehensive prenatal counseling for affected families. Consequently, some families, upon diagnosis of severe fetal anomalies, opted for elective termination of pregnancy before 28 weeks of gestation, contributing to the observed increase in early fetal mortality (30). On the other hand, improvements in prenatal management and neonatal care technologies have enhanced the survival probability of fetuses with structural anomalies, thereby reducing their risk of death in late fetal and early neonatal periods (31–33). The decline in late fetal and early neonatal mortality appeared larger among children born to mothers without Shenzhen household registration, who had higher baseline mortality levels in Phase I.

In line with our findings, international experiences have also highlighted the value of comprehensive congenital anomaly prevention strategies. Postoev et al. assessed the impact of a prenatal ultrasound screening program implemented in 2000 on perinatal mortality due to birth defects in the Kola Peninsula (Northwest Russia), which included 30,448 newborns between 1973 and 2011, reported a decline in perinatal mortality from 21.2‰ during 1973–2000 to 10.0‰ during 2001–2011 (34). Similarly, a population-based cohort study in Northern Netherlands, involving 8,535 fetuses and newborns with congenital anomalies from 1973 to 2011, evaluated the effects of prenatal screening introduced in 2007. The study found a significant increase in therapeutic termination of pregnancy at early gestational ages and in early fetal deaths, while perinatal mortality declined markedly after the implementation of prenatal screening (19). These findings align with our results, indicating that declines in late fetal and early neonatal mortality coincided with the implementation of birth defect prevention and control initiatives. Such temporal associations may contribute to mitigating adverse perinatal outcomes, potentially lessening maternal physical and emotional trauma, as well as the broader social and healthcare burden associated with birth defects (35). The WHO has emphasized integrated approaches combining pre-conception care, folic acid supplementation, prenatal screening, and early intervention as cost-effective public health measures (26, 36). Similarly, analysis for global and developing countries have demonstrated that multiple preventive interventions can reduce the burden of congenital anomalies and improve child survival (37, 38). These global perspectives underscore the importance of developing context-specific, multifaceted prevention programs in China.

The proportion trend of chromosomal/genetic anomalies appears to coincide with the progressive refinement of birth defect prevention programs. During phase II (2018–2021), Shenzhen strengthened the detection of hemoglobinopathies and common chromosomal aneuploidies, when the proportion of chromosomal/genetic anomalies increased from 6.5% to 11.4%. In phase III (2022–2023), with the expansion of screening coverage, the proportion reached 16.9%. In addition, the increasing proportion of advanced maternal age pregnancies, may also explain the rising proportion (39, 40). Throughout the three phrases, early fetal mortality among children with chromosomal/genetic anomalies remained consistently high (ranging from 72.6% to 73.1%), with no statistically significant differences (*P* > 0.05). However, the late fetal mortality decreased from 11.0% to 6.2% (*P*^a^ < 0.001), and early neonatal mortality declined from 1.1% to 0.0% (*P*^a^ < 0.001), with statistically significant differences. These trends may suggest two possibilities: first, prevention and control programs may have contributed to earlier detection and potential intervention for severe chromosomal/genetic anomalies, which could be associated with reduced perinatal mortality risk; second, the broader screening coverage likely increased the detection of milder cases, thereby expanding the number of diagnosed anomalies and potentially helping to stabilize early fetal mortality among children with chromosomal/genetic anomalies.

This study also found that the association between the birth defect prevention and control program and mortality outcomes varied by maternal household registration status. Among the non-local-registered population, notable reductions were observed in late fetal death and early neonatal death across the three phases, whereas changes among the local-registered group were less evident. These patterns may reflect different levels of program relevance between groups. At the same time, higher baseline mortality rates with potential regression to the mean, variations in healthcare-seeking behaviour, access to services, and broader secular improvements in perinatal care may also have contributed to the observed trends. Previous studies have shown that migrant women are more likely to rely on emergency obstetric services rather than preventive care, resulting in lower coverage of routine antenatal check-ups compared with local residents, which in turn delays recognition and intervention for adverse pregnancy outcomes (41, 42). Migrant women also face elevated risks of stillbirth, perinatal, and neonatal mortality, partly due to poorer quality or delayed access to medical services. In some European countries, exclusion of undocumented migrants from free maternity care has been shown to increase the risk of stillbirth and neonatal death by 1.5 to 2 times (43, 44). By extending program coverage to both local and migrant populations, Shenzhen has helped to narrow gaps in antenatal healthcare provision and utilization. For the local-registered population, who already had better access to care and higher health literacy, the additional services may have had limited incremental impact. In contrast, expanded program coverage may have improved access to prenatal care among non-local-registered women, which corresponded with reductions in late fetal death and early neonatal mortality in their children with birth defects.

There are several strengths in this study. Firstly, this study leverages data from the Shenzhen Maternal and Child Health Information System (MCHIS), which includes comprehensive birth defect surveillance data from over 87,000 children over a 12-year period. The large sample size provides robust statistical power to evaluate temporal trends in mortality among children with birth defects, thereby enhancing the reliability of the findings. Secondly, the stratification of data by maternal household registration (local vs. non-local) offers unique insights into the disparities in the effectiveness of policy interventions among different population groups. This analysis provides a nuanced understanding of the program's impact, especially in a rapidly urbanizing, migrant-heavy context. Thirdly, by dividing the study period into three phases, we are able to examine how birth defect mortality rates evolved over time as Shenzhen's birth defect prevention policies were progressively implemented. This longitudinal perspective allows us to track trends and identify the key periods during which policy changes may have had the most significant impact.

However, there are also some limitations. First, due to its retrospective cohort design, causal inferences cannot be definitively drawn. Although significant temporal declines in mortality were observed during the period of program implementation, these trends may also reflect concurrent improvements in maternal and child health services, socioeconomic development, and access to care. Therefore, further prospective or quasi-experimental studies are warranted to more robustly evaluate policy impacts. Second, as the dataset does not clearly distinguish between medical termination and spontaneous miscarriage, we are unable to definitively distinguish between medical termination and spontaneous miscarriage. This limitation hinders a precise attribution of causes underlying the mortality trends. Future research incorporating granular data on specific causes of death and termination indications would be invaluable for further clarifying these complex relationships. Tirdly, due to data limitations, we were unable to obtain more detailed subgroup-specific coverage information for each intervention, as well as income or health insurance coverage. The differences could partly influence the observed outcomes so it is neccessary for us to be more careful in interpreting the subgroup results. Finally, this study focused primarily on early and late fetal death and early neonatal death, but did not assess long-term mortality outcomes, such as infant or childhood mortality, which may lead to an underestimation of its overall effect on survival outcomes. Nevertheless, focusing on perinatal and early neonatal outcomes remains highly relevant.

## 5 Conclusion

In summary, during the implementation period of birth defect prevention and control programs, late fetal and early neonatal mortality in Shenzhen showed a significant decline, particularly among non-local populations. These reductions in mortality coincided with the implementation of the programs, suggesting that the initiatives may have contributed to improved detection and earlier intervention for birth defects, thereby potentially reducing the burden on affected families and society. While causality cannot be established, the observed temporal associations underscore the importance of sustaining comprehensive prevention and control programs. For policymakers in other rapidly urbanizing regions, Shenzhen's experience may provide valuable insights for designing integrated maternal and child health strategies.

## Data Availability

The raw data supporting the conclusions of this article are available from the corresponding author upon reasonable request.
